# Pathways Linking Nicotinamide Adenine Dinucleotide Phosphate Production to Endoplasmic Reticulum Protein Oxidation and Stress

**DOI:** 10.3389/fmolb.2022.858142

**Published:** 2022-05-04

**Authors:** Erica R. Gansemer, D. Thomas Rutkowski

**Affiliations:** ^1^ Department of Anatomy and Cell Biology, Carver College of Medicine, University of Iowa, Iowa City, IA, United States; ^2^ Department of Internal Medicine, Carver College of Medicine, University of Iowa, Iowa City, IA, United States

**Keywords:** endoplasmic recticulum, metabolism, redox, glutathione, nicotinamide adenine dinucleotide phosphate

## Abstract

The endoplasmic reticulum (ER) lumen is highly oxidizing compared to other subcellular compartments, and maintaining the appropriate levels of oxidizing and reducing equivalents is essential to ER function. Both protein oxidation itself and other essential ER processes, such as the degradation of misfolded proteins and the sequestration of cellular calcium, are tuned to the ER redox state. Simultaneously, nutrients are oxidized in the cytosol and mitochondria to power ATP generation, reductive biosynthesis, and defense against reactive oxygen species. These parallel needs for protein oxidation in the ER and nutrient oxidation in the cytosol and mitochondria raise the possibility that the two processes compete for electron acceptors, even though they occur in separate cellular compartments. A key molecule central to both processes is NADPH, which is produced by reduction of NADP+ during nutrient catabolism and which in turn drives the reduction of components such as glutathione and thioredoxin that influence the redox potential in the ER lumen. For this reason, NADPH might serve as a mediator linking metabolic activity to ER homeostasis and stress, and represent a novel form of mitochondria-to-ER communication. In this review, we discuss oxidative protein folding in the ER, NADPH generation by the major pathways that mediate it, and ER-localized systems that can link the two processes to connect ER function to metabolic activity.

## Introduction—Why Should the Endoplasmic Reticulum Care What Is Happening in the Mitochondria?

The extensive physical contacts between the ER and mitochondria underscore the need for these two organelles to communicate. Why is that communication necessary? At the simplest, level, the ER requires ATP to fold and transport nascent secretory and membrane proteins. Yet beyond ATP output, it is likely that metabolic activity within the mitochondria will profoundly impact ER function in both direct and indirect ways, even though the mitochondrial matrix is compartmentally separated from the ER lumen by three membranes—the ER membrane and the mitochondrial inner and outer membranes. The tricarboxylic acid (TCA) cycle is the central hub of metabolism; carbohydrates and lipids catabolized to acetyl-CoA enter the cycle by condensation with oxaloacetate to form citrate, and one turn through the cycle generates NADH and FADH_2_ for oxidative phosphorylation. But, importantly, the TCA cycle is much more than a simple means to ATP production. TCA flux drives biosynthetic pathways such as lipogenesis, gluconeogenesis, and ketogenesis, the first two of which take place either in whole or in part, respectively, at the ER. Some of these biosynthetic pathways—including lipogenesis—are reductive in nature, requiring not just precursors from the TCA cycle such as citrate, but also NADPH produced either through TCA cycle isozymes or other metabolic pathways such as the pentose phosphate pathway (PPP) and one-carbon (1C) metabolism. NADPH also maintains cellular redox homeostasis, and as will be discussed below, proper oxidation and reduction of proteins in the lumen of the ER is central to homeostasis there. Thus, the simple flow of nutrients into the mitochondria stimulates biochemical processes that directly impact ER function. Moreover, exposure to nutrients and insulin that accompany a meal also drives protein biogenesis through the activation of mTOR signaling (Oyadomari et al., 2006; Pfaffenbach et al., 2006). Because, in a typical cell, secretory and membrane proteins—i.e., ER “client proteins”—comprise approximately one-third of the total cellular proteome , increased nutrient catabolism portends an imminent demand on the ER biosynthetic capacity. Thus, it stands to reason that the ER will be responsive to metabolic flux and TCA cycle activity in ways beyond just the production of ATP.

One of the unique features of ER protein folding, compared to cytosolic protein folding, is the need to oxidize proteins in the form of disulfide bonds on cysteine residues. Because of this need, the environment of the ER lumen is net-oxidizing toward its client proteins, and an inability to form disulfide bonds would be expected to dramatically compromise ER function. In support of this idea, reducing agents such as dithiothreitol are among the most potent elicitors of ER stress. Yet ER client protein oxidation must be tightly regulated, because over-exuberant oxidation can cause the formation of disulfide-bonded protein aggregates, and can also prevent proteins from achieving the correct disulfide bonded configuration, for which the ability to reduce and reform (i.e., isomerize) disulfide bonds is needed.

Our central thesis is that the generation of reducing equivalents by metabolic activity directly links ER homeostasis to nutrient intake and catabolism. In other words, it is likely that the central functions of the ER—particularly protein folding and secretion and calcium storage—wax and wane with feeding and fasting cycles because of the effects of NADPH on the oxidative environment within the ER. Yet intermediary metabolism and ER homeostasis, though interconnected processes, are disparate fields. Thus, one goal of this review is to sketch the pathways of NADPH production to the ER expert and the pathways of oxidative protein folding to the metabolism expert. Then, we will review the most likely candidates for conveying changes in the cellular NADPH status from the mitochondria and cytosol to the ER lumen, and we will highlight ER functions that are likely to be particularly sensitive to such changes. Along the way, we will highlight instances when these basic biochemical pathways are linked to obesity, and speculate about the potential impacts of metabolic activity on ER function.

## The Endoplasmic Reticulum and Endoplasmic Reticulum Stress

As gateway to the secretory pathway, one of the most important functions of the ER is the synthesis, folding, modification, and trafficking of secretory proteins and resident proteins of the endomembrane system. ER stress arises when there is imbalance between the load of ER client proteins that must be processed and the ability of the organelle to properly process them. Oxidative protein folding, which is carried out by a dedicated system of oxidases and thiol isomerases in the ER lumen, is coupled to the translocation of proteins across the ER membrane, their modification by N-linked glycans, and their association with ER-resident chaperones.

### Protein Biogenesis and Oxidation

Canonically, secretory and transmembrane proteins are targeted to the ER membrane by the signal recognition particle (SRP) which recognizes both cleavable N-terminal signal peptides and internal non-cleaved transmembrane domains. Engagement of the translocon by the targeted nascent polypeptide opens a channel into the ER lumen, into which the polypeptide emerges (Nyathi et al., 2013). Protein folding and covalent modification begin immediately when the nascent polypeptide gains access to the lumen, meaning these processes are tightly coupled. Cotranslational modifications include disulfide bond formation—which will be discussed in detail—signal peptide cleavage, and N-linked glycosylation (Braakman and Bulleid, 2011; Ellgaard et al., 2016). These modifications are functionally interdependent because they rely on accessibility of specific structural features—cysteines, signal peptide cleavage sites, and Asn-(not Pro)-Ser/Thr, respectively—by discrete ER machinery (Ellgaard et al., 2016). For example, signal peptide cleavage usually occurs cotranslationally, but can vary from an early event in the synthesis and translocation of the polypeptide—occurring almost immediately after the polypeptide accesses the ER lumen—to a much later one, even in some cases occurring posttranslationally. Prior to its removal, the signal peptide can alter the conformation and local environment of the nascent polypeptide and the ER lumenal and membrane factors to which it has access (Daniels et al., 2003; Rutkowski et al., 2003). In perhaps the most notable case, the signal peptide of the HIV-1 envelope glycoprotein, gp160, is not cleaved until at least 15 min after its synthesis has been completed (Land et al., 2003). Mutating its signal peptide to accelerate its cleavage alters the timing of gp160 oxidation, the final conformation of the gp160 protein, and ultimately the relative fitness of the virus (Snapp et al., 2017). Likewise, oxidation and N-glycosylation can be competing processes (Allen et al., 1995; Holst et al., 1996), and N-glycosylation also allows nascent proteins to access ER-resident calnexin/calreticulin lectin proteins that bring misfolded nascent glycoproteins into proximity with thiol isomerases for disulfide bond rearrangement (Caramelo and Parodi, 2008). Thus, though the ER lumen is oxidizing toward nascent proteins, oxidation is a highly contingent process, and disruption to other pathways of ER folding and modification are likely to have knock-on effects on disulfide bond formation and rearrangement.

The extensive formation of disulfide bonds is a feature relatively unique to the ER folding environment of eukaryotic cells, with only the mitochondrial intermembrane space also having dedicated protein oxidation machinery (Riemer et al., 2009). Disulfide bonds form by a two-electron reaction that moves electrons from reduced sulfhydryls in the protein to an oxidizing equivalent. Most ER proteins are oxidized, some extensively, and thus oxidizing equivalents must be abundant in the ER lumen for folding to occur efficiently. Because the ER lumen is oxidizing, disulfides begin to form co-translationally as soon as reactive cysteines emerge into the ER (Chen et al., 1995; Bulleid, 2012; Ellgaard et al., 2016). However, the disulfide bonds needed for correct protein conformation are often not sequentially oriented on the polypeptide, and thus non-native disulfides can form that must be subsequently reduced and isomerized for the protein to be correctly folded. As a result, the ER lumen needs to be able to both oxidize and reduce disulfides.

**FIGURE 1:**
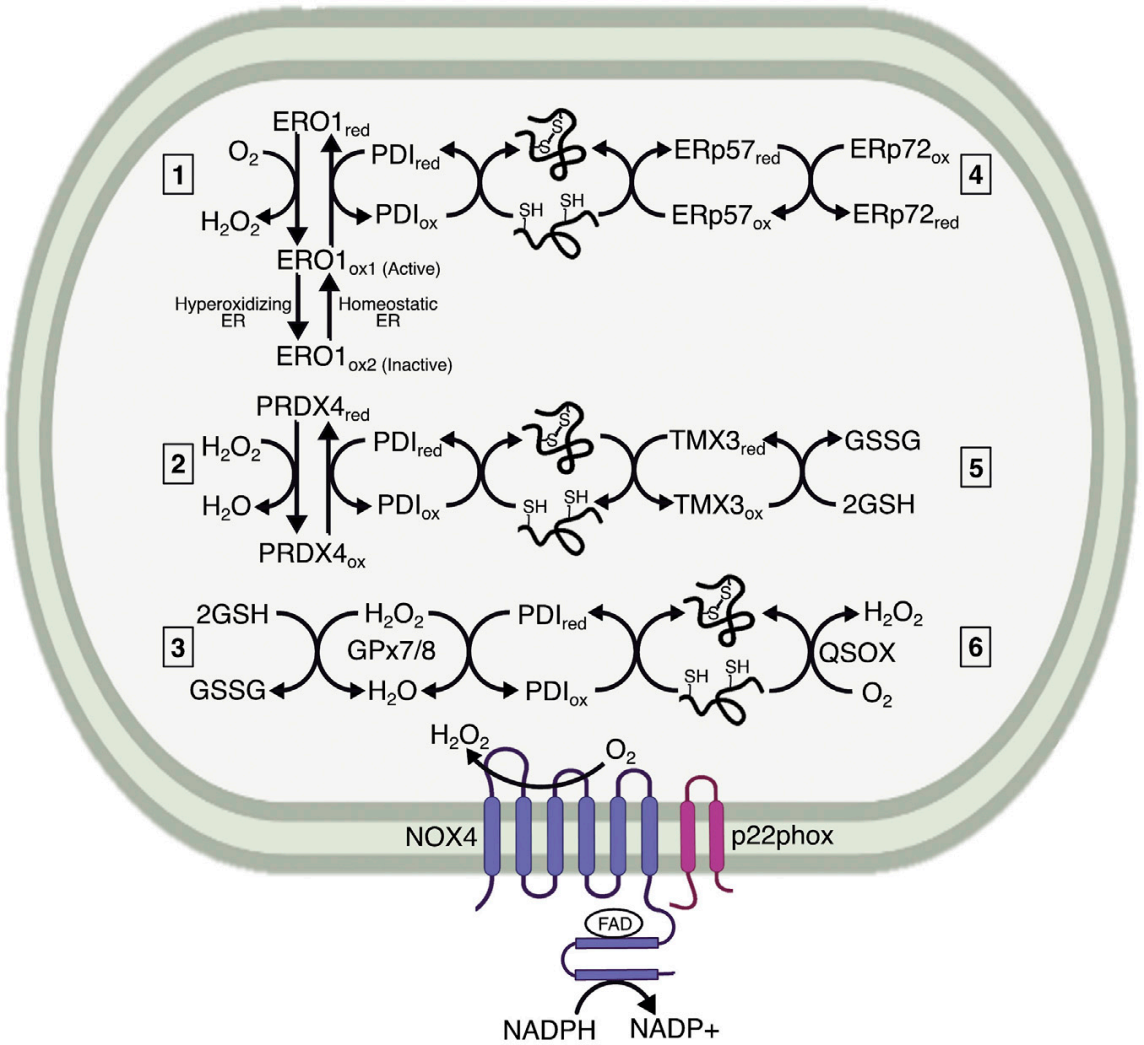
Mechanisms maintaining the oxidative capacity of the ER lumen. The ER lumen must be sufficiently oxidizing to maintain protein folding. It is characterized by a high preponderance of GSSG, which is generated by ER-localized GPx7/8 as a method of neutralizing H_2_O_2_. The extent to which GSSG influences protein folding remains less understood. The main ER resident proteins controlling oxidative folding are the PDI family members. PDIs are maintained in an oxidized state by electron transfer to 1) ERO1, generating H_2_O_2_ in the process; 2) PRDX4, which neutralizes H_2_O_2_; and 3) GPx7/8, which also neutralizes H_2_O_2_. Both PRDX4 and GPx7/8 likely use H_2_O_2_ generated by ERO1 or NADPH oxidase 4 (NOX4) to reoxidize PDI. ERO1 also undergoes redox-mediated regulation by existing in two oxidized states, indicated here as ERO1_ox1_ and ERO1_ox2_, wherein the ox1 state has oxidized catalytic cysteines and reduced regulatory cysteines, and is therefore active, versus ox2, which has oxidized regulatory cysteines and is therefore inactive. The ox1 form predominates under normal ER lumenal conditions, whereas the ox2 form is generated with the ER lumen becomes hyperoxidizing. Additional interactions are proposed between PDI family members, represented here by ERp57 (PDIA3) and ERp72 (PDIA4). 4) These interactions could be involved with both oxidation for initial disulfide formation and reduction for disulfide isomerization. Some PDIs, such as TMX3, are proposed to interact directly with GSH, which reduces PDI for disulfide reduction and isomerization. 5) Finally, a potential mechanism for protein oxidation catalyzed by QSOX, 6) which transfers electrons to oxygen and generates H_2_O_2_. The extent to which each of these mechanisms affects protein processing has not been fully elucidated.

Oxidation and isomerization are performed by thiol isomerases. To date, approximately 20 proteins are classified as thiol isomerases, the exemplar of which is protein disulfide isomerase (PDI) (Ellgaard and Ruddock, 2005), underscoring the importance of disulfide bond catalysis. PDI consists of four domains (*a, b, b’, a’*), two of which (*a* and *a’*) contain thioredoxin-related reactive CXXC motifs that facilitate disulfide exchange (Chivers et al., 1997). The *b* and *b’* domains are not catalytically active, but act as chaperones for substrate binding (Klappa et al., 1998). The catalytic *a* and *a’* domains are sufficient for protein oxidation, but the *b* and *b’* domains are necessary for isomerization (Darby et al., 1996). Oxidized PDI serves as an electron acceptor during disulfide catalysis, whereas reduced PDI serves as an electron donor during reduction and isomerization. Reoxidation of PDI is essential for its function, and was initially thought to rely on oxidized glutathione (GSSG). However, depletion of ER glutathione minimally impacts PDI (Cuozzo and Kaiser, 1999; Tsunoda et al., 2014), and further work identified ER oxidoreductase 1-alpha (ERO1α), ER-localized peroxiredoxin 4 (PRDX4), glutathione peroxidase 7 (GPx7), glutathione peroxidase 8 (GPx8), and Vitamin K epoxide reductase as alternative mechanisms for PDI oxidation (Cabibbo et al., 2000; Wilkinson and Gilbert, 2004; Zito et al., 2010; Nguyen et al., 2011; Rutkevich and Williams, 2012) (Figure 1). However, it is worth noting that PDI reacts extremely efficiently with GSSG, and that the sheer abundance of GSSG in the ER lumen—by one estimate, 2 mM—might leave a role for this molecule in PDI reoxidation (Lappi and Ruddock, 2011), especially in certain cell types, like hepatocytes, in which glutathione is particularly abundant (Forman et al., 2009). PDIs can also react with GSH during thiol isomerization (Appenzeller-Herzog and Ellgaard, 2008). GSH is reportedly required for reduction of ER protein 57 (ERp57, PDIA3) (Jessop and Bulleid, 2004), and may directly equilibrate with thioredoxin-related transmembrane protein 3 (TMX3) (Haugstetter et al., 2005). Additionally, PDI family members engage in disulfide exchange with each other, whether to promote oxidative folding or isomerization. For example, ERp57 can be oxidized by ER-protein 72 (ERp72, PDIA4) to promote disulfide bond catalysis (Oka et al., 2015). Ultimately, the relative contribution of each of these pathways of PDI reoxidation and reduction under different cellular conditions remains unclear.

ERO1 was initially identified in yeast as an essential protein for disulfide formation (Frand and Kaiser, 1998; 1999). There are two mammalian homologs, ERO1α, and ERO1β. ERO1α is widely expressed, whereas ERO1β is enriched in the pancreas and immunoglobulin-producing cells (Dias-Gunasekara et al., 2005), both of which are highly secretory cells, suggesting that the need for oxidation may correlate with secretory load. ERO1 is an ER-resident N-glycoprotein that does not contain an ER retention signal (KDEL), but is retained in the ER nonetheless. Subsequent work identified covalent interactions between ERO1α and PDI, as well as the PDI family member, ER-protein 44 (ERp44) (Otsu et al., 2006). The interaction with ERp44 is essential for ERO1 retention, whereas the interaction with PDI prevents ERO1 dimerization and aggregation (Otsu et al., 2006). ERO1 is likely aggregation-prone based on its structure: it contains an outer thioredoxin-like CXXCXXC motif that is catalytically active, an inner C-terminal active site near an FAD moiety, and 12 additional cysteine residues. The active sites are organized into loops, with the outer loop being flexible enough to “shuttle” electrons to the inner loop. To re-oxidize PDI, the outer disulfide in ERO1 accepts electrons from PDI and shuttles them to the inner active site and FAD motif (Tu et al., 2000; Tu and Weissman, 2002). Subsequently, electrons are transferred to molecular oxygen—generating H_2_O_2_—to maintain ERO1 in an oxidized state.

The additional cysteines in ERO1 can also form intramolecular disulfides that serve regulatory roles. In particular, two disulfides form that connect the outer and inner loops, which affects the flexibility of the outer loop. For this reason, these residues are considered regulatory cysteines that act as a redox-regulated switch to control ERO1 (Sevier et al., 2007; Appenzeller-Herzog et al., 2008), such that highly oxidizing conditions in the ER lumen lead to formation of an intramolecular disulfide bond that inactivates ERO1 enzymatic activity and prevents overexuberant oxidation. The rapid re-oxidation of ERO1 is also a mechanism to limit ERO1 activity. By generating H_2_O_2_, ERO1 creates hyperoxidizing microdomains that favor oxidation of the regulatory cysteines in ERO1, and prevent its activity (Sevier et al., 2007). This regulation and inactivation is important because hyperactive ERO1 is a source of oxidative damage (Hansen et al., 2012). In this way, the cell can maintain tight control over protein oxidation. Paradoxically, it is possible that stimuli that make the ER more oxidizing could actually lead to diminished oxidation of ER proteins due to this negative feedback loop. Overexpression of a constitutively active ERO1 mutant incapable forming this regulatory disulfide elicits ER stress, but to a relatively modest extent compared with reducing agents like DTT (Hansen et al., 2012; Eletto et al., 2014a).

While ERO1 has been suggested to be the major regulator of PDI redox cycling, it is not required for disulfide formation in mammals, which prompted identification of other redox regulators in the ER. Use of a PDI trapping mutant identified PRDX4 as a candidate for oxidizing PDI and restoring protein folding (Zito et al., 2010). PRDX4 is a 2-cysteine peroxiredoxin that was identified as a soluble ER lumenal protein (Tavender et al., 2008). The peroxiredoxins are widely expressed H_2_O_2_ scavengers containing a highly conserved PXXT/SXXC motif, which is conjugated to sulfenic acid when neutralizing H_2_O_2_, and then forms an intra- or intermolecular disulfide bond (Perkins et al., 2015). The ability of peroxiredoxins to form intermolecular disulfides led to the hypothesis that PRDX4 could form a complex with, and re-oxidize, PDI. PRDX4 does oxidize PDI, and accelerates PDI’s ability to use H_2_O_2_ as an electron acceptor during protein folding (Zito et al., 2010). Additionally, because H_2_O_2_ produced by ERO1 preferentially oxidizes PRDX4 when ERO1 is highly active (Tavender and Bulleid, 2010), ERO1 could accelerate PRDX4-mediated protein folding.

Similar to PRDX4, GPx7 can use H_2_O_2_ produced by ERO1 to accelerate protein folding (Wang et al., 2014). GPx7 is an atypical two-cysteine GPx because it lacks a canonical resolving cysteine commonly needed for reduction during catalytic cycles (Toppo et al., 2008; Nguyen et al., 2011). The first cysteine in GPx7 is peroxide-reactive, whereas the second acts as a resolving cysteine, creating an intramolecular disulfide bond when the first cysteine is sulfenylated. Additionally, sulfenylated and disulfide bonded GPx7 can interact with the catalytic domains of PDI. Interestingly, GPx7 and ERO1 can interact with PDI simultaneously, with ERO1 oxidizing the *a’* domain of PDI, and generating H_2_O_2_ that is subsequently used by GPx7 to oxidize the *a* domain (Wang et al., 2014). The consumption of H_2_O_2_ by PRDX4 and GPx7 is beneficial because it prevents oxidative stress (Tavender and Bulleid, 2010; Kim et al., 2020). Though the protection is localized to the ER, it potentially extends to the cytosol by preventing consumption of reduced glutathione (discussed below), which is a major cytosolic and mitochondrial antioxidant.

Though PDI-mediated oxidation predominates, there are proposed mechanisms of protein oxidation that do not require PDI. The main proteins responsible are sulfhydryl oxidases, which often incorporate iron, copper, or flavin binding domains to participate in oxidation-reduction reactions (Kodali and Thorpe, 2010). The flavoprotein—quiescin sulfhydryl oxidase (QSOX)—functions similarly to ERO1, but interacts directly with reduced thiols instead of employing PDI. The interaction with reduced thiols is achieved through a thioredoxin domain similar to that in PDI (Kodali and Thorpe, 2010). Due to the localization of QSOX in the Golgi and secretory granules, its function may be to ensure disulfide integrity during late-stage maturation and secretion, as well as formation of larger complexes after secretion (Chakravarthi et al., 2007). However, whether QSOX plays a role in protein folding in the ER remains less understood.

As a whole, oxidative folding—whether forming or reducing disulfides—constitutes a major burden for the cell, particularly in cell types with high secretory loads. A classic example is hepatocytes which secrete a high volume of albumin—by far the most abundant component of plasma—along with lipoproteins, clotting factors, and antiproteases. Albumin itself has 17 disulfide bonds (Dugaiczyk et al., 1982; Sugio et al., 1999), meaning the ER in hepatocytes must maintain a high capacity for forming disulfide bonds. In addition, because albumin is the most abundant ER client protein in hepatocytes, and its disulfide bonds are largely sequential, the hepatocyte ER might have less of a need for disulfide reduction, though this idea has not been tested. Likewise, correct disulfide bonding of Apolipoprotein B, which is the major protein component of very low density lipoprotein (VLDL) produced by the liver, is required for VLDL particle assembly in the ER (Shelness and Thornburg, 1996). VLDL has seven disulfide bonds, the first two of which are overlapping and the remaining five of which are sequential. VLDL production is induced under conditions of nutrient sufficiency and suppressed during fasting (Wilcox and Heimberg, 1987; Choi and Ginsberg, 2011). Therefore, it might be expected that hepatocytes have a keen need for oxidizing equivalents in the ER generally, and even more so during the latter stages of nutrient excess. Conversely, pancreatic beta cells must reduce a disulfide bond in pro-insulin for proper insulin maturation, packaging, and secretion, and insulin biogenesis is stimulated by glucose metabolism (Fu et al., 2013). Thus, beta cells might have a greater requirement for ER reducing power. In support of this idea, proinsulin maturation was impaired in beta cells with a hyperoxidizing ER (Rohli et al., 2021). Therefore, it is conceivable that different cell types, such as hepatocytes and beta cells, have distinct needs for oxidation versus reduction, dependent not only on the types of proteins that a given cell type has to secrete, but also on cellular conditions such as nutrient fluxes that impact the relative composition of the secretome at any given time. Much of our understanding of the cellular need for oxidizing versus reducing equivalents in the ER has come from studies in a limited sampling of cell types such as HEK293 and mouse embryonic fibroblasts that might or might not be universally applicable. Determining the basal capacity of different cell types to oxidize and reduce proteins, and their relative responsiveness to hyper- and hypo-oxidation, will increase our understanding of how cellular redox requirements affect ER function.

### Assessing the Endoplasmic Reticulum Redox State

While the ER has long been recognized as an oxidizing environment capable of *de novo* formation of disulfide bonds on nascent proteins, only the development of ER-localized redox-sensitive modified GFPs has enabled non-invasive measurement of the ER redox potential and its responsiveness to perturbations. Most such sensors are based on roGFP (for reduction-oxidation-sensitive GFP), which has two surface residues mutated to cysteines to allow for formation of a disulfide bond under oxidizing conditions (Hanson et al., 2004). The disulfide bond shifts the excitation maximum for the GFP relative to the reduced protein, thus creating a ratiometric sensor. Unfortunately, as useful as roGFPs were in detecting oxidative shifts in the cytosol and mitochondrial matrix, the stability of the disulfide bond made them ill-suited to measuring redox alterations in the oxidizing environment of the ER. To address this issue, further mutations were introduced to destabilize the roGFP disulfide bond (Lohman and Remington, 2008). These modified roGFPs were used to show responsiveness of ER redox to ERO1 manipulation (Van Lith et al., 2011) and to application of ER stress-inducing agents (Avezov et al., 2013). Fusion of the ER-targeted construct to glutaredoxin gave the readout specificity for glutathione (Birk et al., 2013) just as it does to roGFP in the cytosol (Gutscher et al., 2008). However, imaging with these roGFPs was difficult because GFP folds very inefficiently in the ER (Costantini and Snapp, 2013). Application of the roGFP surface mutations on superfolder GFP (sfGFP), which fluoresces brightly even in oxidizing environments (Pédelacq et al., 2006; Aronson et al., 2011) has more recently allowed for an ER-localized roGFP that is bright and has an appropriate dynamic range for assessing ER redox (Hoseki et al., 2016). Notably, the challenges of creating biosensors that function properly in the ER are not limited to roGFP. HyPer, which in the cytosol is ratiometric sensor for H_2_O_2_ that is based on circularly permuted GFP, in the ER is only a more generic readout for ER redox poise (Mehmeti et al., 2012).

### Endoplasmic Reticulum Stress and Metabolism

Stimuli that compromise the capacity of the ER to fold and export client proteins are categorized as ER stressors. Most evidently, ER stress is induced by deletion of ER chaperones, overexpression of constitutively misfolded proteins, and disruption of N-linked glycosylation, disulfide bond catalysis, or calcium homeostasis. Other stimuli, including loading with saturated fatty acids, which perturbs membrane fluidity, elicit ER stress despite less obvious connections to protein folding. With the importance of disulfide bond formation in the ER having long been appreciated, reducing agents such as DTT were among the first chemicals used to characterize the ER stress response (Whelan and Hightower, 1985). ER stress is sensed by the unfolded protein response (UPR) (Ron and Walter, 2007). The canonical UPR pathways—inositol-requiring enzyme 1α (IRE1α), protein kinase RNA-like ER kinase (PERK), and activating transcription factor 6 (ATF6)— activate transcription of ER chaperones to help clear misfolded proteins, whether through re-folding or shuttling them for ER-associated degradation (ERAD). The UPR also suppresses translation of ER client proteins to decrease the overall protein load in the ER, induces autophagy to assist in protein degradation (Yorimitsu et al., 2006), and activates regulated mRNA degradation to diminish protein influx (Hollien et al., 2009). The UPR elicits both cytoprotective and cytotoxic signaling cascades, with the latter including cell death and inflammation [reviewed in (Iurlaro and Muñoz-Pinedo, 2016)].

UPR activation is readily assessed by several readouts, and is often used synonymously with “ER stress,” the latter of which is difficult to measure directly. While it is clear that pharmacological stressors that grossly perturb ER function also result in robust UPR activation, the conflation of ER stress and UPR activation obscures the fact that the UPR may not respond equally to all ER perturbations. For instance, disrupting disulfide bond formation with reducing agents has long been accepted as an extremely robust activator of the UPR. Conversely, the opposite—a hyperoxidizing ER—elicits comparatively modest UPR activation despite impairing protein maturation and preventing disulfide isomerization (Hansen et al., 2012). Thus, even though it is important from the point of view of client protein function that the ER retain enough reducing power to prevent protein hyperoxidation, it does not necessarily portend that an abundance of hyperoxidized proteins are sensed as a disruption to ER homeostasis as readily as an abundance of underoxidized proteins. Indeed, while it is widely accepted that ER stress arises when misfolded proteins accumulate in the ER, it is not currently clear whether all potential forms of misfolding—such as hydrophobic-driven aggregation, underoxidation, overoxidation, impaired N-glycosylation, etc.—are perceived as equally “stressful” to the organelle. While UPR activation diagnoses the presence of at least transient ER stress, and the terms are used somewhat interchangeably even here, its absence does not necessarily eliminate the possibility that disruptions to ER homeostasis have occurred or are occurring.

UPR activation is linked with obesity in several tissues, implying that metabolic activity, or the dysregulation thereof, impacts ER homeostasis. UPR activation is evident in obese animals and humans in several tissues, including the brain, liver, muscle, and adipose (Sharma et al., 2008; Kawasaki et al., 2012; Horwath et al., 2017). Indeed, obesity causes a drastic disruption to and rearrangement of ER morphology in hepatocytes (Parlakgül et al., 2022). However, it remains unclear how obesity leads to ER stress. Among previously suggested pathways are lipotoxicity arising from ectopic accumulation of fat in tissues other than adipose, such as liver and muscle (Mantzaris et al., 2011; Lake et al., 2014; Patterson et al., 2016) and disruption of ER calcium homeostasis (Fu et al., 2011). However, the root of obesity is overnutrition, which involves frequent, excessive caloric intake. Therefore, an additional possibility is that catabolism of nutrients *per se*, could contribute to ER stress, and that obesity is simply a compounding of this effect, applied across many meals over many years. In support of this idea, feeding after a fast in mice is sufficient to induce ER stress in the liver. This effect is independent of the fat content of the meal, suggesting that it is the consumption of calories rather than fat specifically that drives the effect (Gomez and Rutkowski, 2016). The ER stress arising from feeding has been proposed to be driven in part by mTOR and the consequent upregulation of protein synthesis, which would be expected to increase the load of client proteins entering the ER (Ozcan et al., 2008; Pfaffenbach et al., 2010).

In addition to these potential pathways, we recently discovered that ER homeostasis is linked to TCA cycle activity, and blocking entry of fats or carbohydrates into the TCA cycle diminishes the sensitivity of metabolically active cells like hepatocytes, myocytes, and adipocytes to ER stress; conversely, driving substrate entry into the TCA cycle causes ER stress, to an extent nearly as robust as the bona fide ER stressor tunicamycin (Gansemer et al., 2020). These findings suggest that a key previously unappreciated link between overnutrition and ER stress might be the flux of nutrients through mitochondrial metabolic pathways. In principle, this link might be conveyed by any output of mitochondrial activity, and, indeed, recent work suggests that exchange of calcium, reactive oxygen species (ROS), and potentially other metabolites between mitochondria and the ER likely influences metabolic disease progression (Arruda et al., 2014; Tubbs et al., 2014; Theurey et al., 2016; Theurey and Rieusset, 2017; Tubbs et al., 2018). However, cellular redox—more specifically the linkage of nutrient oxidation to NADPH production—is emerging as an additional potential candidate for transmitting nutrient signals to the ER (Gansemer et al., 2020; Van Lith et al., 2021).

Given that both catabolism and secretion require oxidation—of nutrients in the mitochondria and nascent proteins in the ER—it is plausible that the two pathways compete with each other for electron acceptors. But several questions arise: How do cells balance the need for nutrient and protein oxidation? Does overnutrition induce subcellular redox changes that affect protein homeostasis? Are nutrient and protein oxidation inherently incompatible due to redox limitations? That the UPR decreases lipid oxidation, and that this decrease attenuates ER stress sensitivity (Tyra et al., 2012; DeZwaan-McCabe et al., 2017), supports the idea of incompatibility between nutrient and protein oxidation, though further characterization in nutrient replete and depleted conditions is needed.

## Production of Reduced Nicotinamide Adenine Dinucleotide Phosphate

**FIGURE 2:**
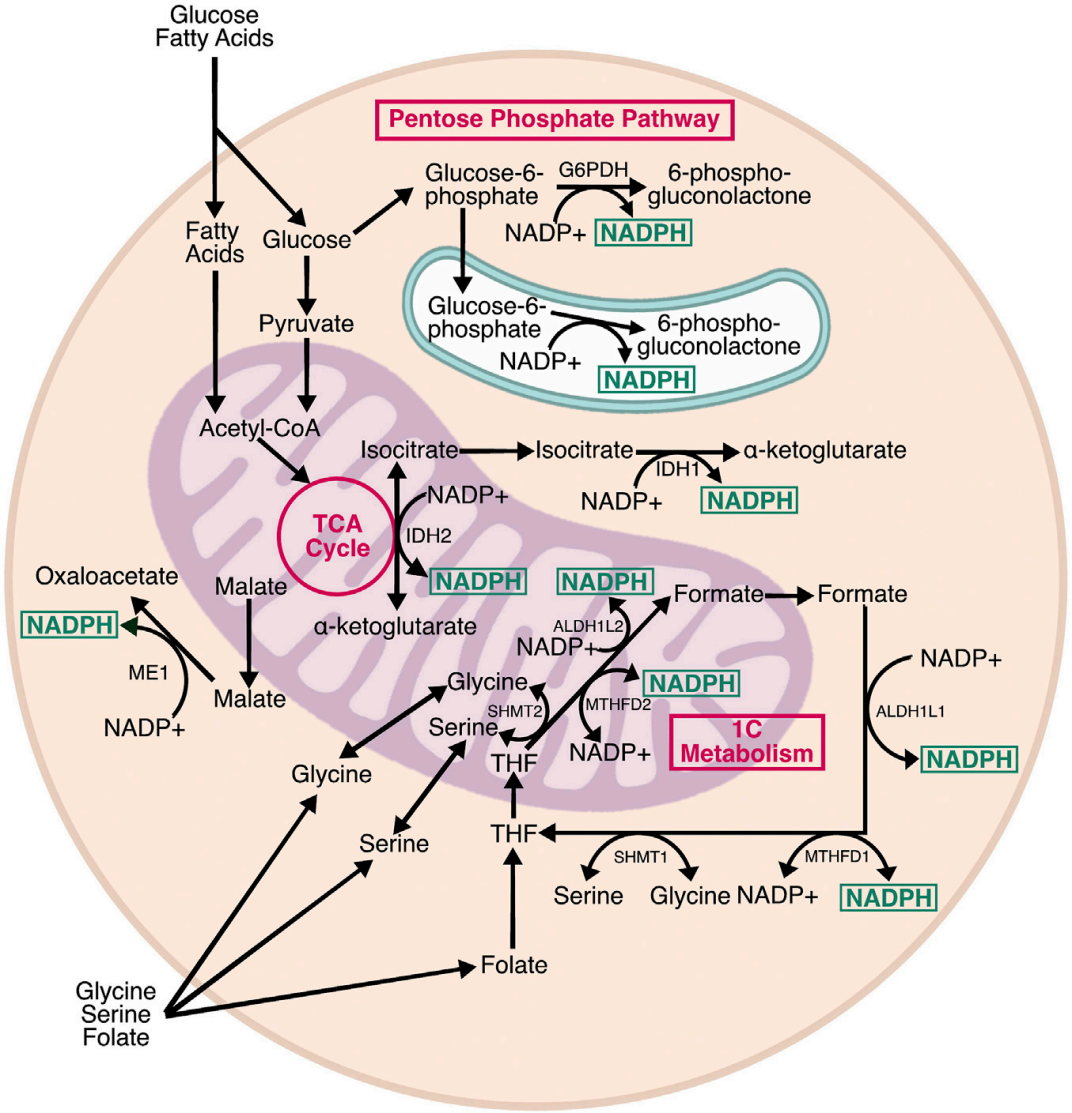
Pathways producing NADPH. Cells contain three subcellular pools of NADPH. In the cytosol, NADPH is produced by G6PD, the rate limiting step of the pentose phosphate pathway, as well as IDH1 and ME1, which are isozymes associated with the TCA cycle. In the mitochondria, NADPH is produced by IDH2, a TCA cycle isozyme, and two enzymes associated with one carbon (1C) metabolism: ALDH2L1 and MTHFD2. IDH1, IDH2, and MTHFD2 can proceed in the reverse direction, consuming NADPH in the process. MTHFD2 can also use NAD+ as a cofactor and generate NADH. In the ER, H6PD—a homolog of G6PD—generates NADPH from glucose-6-phosphate. The relative contribution of each pathway varies by cell type, and there is evidence of crosstalk between NADPH-producing enzymes to maintain optimal NADPH levels.

NADPH is a central mediator of cellular redox homeostasis in multiple cellular compartments, and also an essential driver of reductive biosynthetic pathways. Metabolically, NADPH is generated by three pathways: the pentose phosphate pathway (PPP), the tricarboxylic acid (TCA) cycle, and folate/one-carbon metabolism (Figure 2). The PPP and TCA cycle contribute to cytosolic NADPH, whereas mitochondrial NADPH is generated by the TCA cycle and folate metabolism. There is also an NADPH pool in the ER, which is generated by a branch of the PPP. In the mitochondria, NADPH provides reducing power to neutralize reactive oxygen species (ROS) produced by nutrient oxidation. In the cytosol, NADPH is mainly used for reductive biosynthesis of lipids, cholesterol, and steroids, but also provides reducing power to neutralize ROS that are exported from the mitochondria and ER. In the ER, NADPH indirectly provides reducing power for disulfide isomerization, and contributes to steroid activation (Atanasov et al., 2004; Rogoff et al., 2010). Interestingly, there are no known transmembrane transporters for NADP+ or NADPH, meaning the relative pools in each subcellular compartment are likely generated and consumed locally.

Understanding the regulation of NADPH has been greatly enhanced by the development of compartment-specific probes for monitoring and manipulating the NADPH/NADP+ ratio. Traditional approaches for measuring NADPH in specific cellular compartments relied on cell separation and biochemical discrimination of the redox pair by various methods. However, these approaches were inherently limited by the difficulty in assuring that a compartmental separation was sufficiently pure, and by the ready tendency of NADPH to oxidize during sample preparation. These limitations were overcome by circular permutation—i.e., swapping the N- and C-termini and separating them by a linker—of YFP. This manipulation maintains the chromophore maturation ability of the fluorescent protein but relaxes the structure relative to native YFP and its relatives, such that the site of chromophore maturation is more accessible to environmental influence (Kostyuk et al., 2019). A biosensor for NADPH, called iNap, was developed by mutation of an existing chimera known as SoNar—in which the NADH-binding domain of the Rex protein from *T. aquaticus* (Zhao et al., 2015) was fused to cpYFP—to generate NADPH specificity (Tao et al., 2017). The binding of NADPH by this construct shifts the excitation wavelength of the YFP from 485 to 420 nm. A modified iNap fused to a mitochondrial targeting peptide allows for assessment of intra-mitochondrial NADPH. In both cases, an NADPH-unresponsive control construct is needed to control for the generic effects of pH changes on cpYFP fluorescence. No ER-localized iNap has yet been reported, likely because the highly oxidizing environment of the ER places traditional GFP-based biosensors well outside their redox-sensitive dynamic range (Costantini and Snapp, 2013). Recently, a different probe for NADPH/NADP+, called NADP-Snifit, was created in which SNAP and Halo fluorophores were tethered to the NADP-dependent sepiapterin reductase (SPR) protein, which reads out on NADP+-versus-NADPH binding by virtue of SPR permitting or preventing, respectively, FRET between SNAP and Halo (Sallin et al., 2018). The general approach of utilizing NADP(H)-binding proteins was also harnessed to allow for compartment-specific modulation of the NADPH/NADP+ ratio. In this case, the NADH oxidase from *L. brevis* [LbNOX (Titov et al., 2016)] was rationally mutated to confer NADPH specificity, in a construct named TPNOX (Cracan et al., 2017). Mitochondrial and cytosolic expression of TPNOX was used in HeLa cells to demonstrate that mitochondrial production of NADH was sensitive to disruption of mitochondrial NADPH, while cytosolic NADH was not analogously responsive. These findings pointed to the importance of mitochondrial-specific pathways for exchanging reducing equivalents between the two nucleotides.

### The Pentose Phosphate Pathway

The PPP is thought to be the primary source of cytosolic NADPH—although whether this is true for all cell types and circumstances is less clear—and is required to support the basal NADPH/NADP ratio. The PPP is a glycolytic shunt with oxidative and non-oxidative branches (Eggleston and Krebs, 1974). The oxidative branch generates NADPH for reductive biosynthesis, and ribose-5-phosphate for purine and pyrimidine metabolism, whereas the non-oxidative branch incorporates several glycolytic intermediates to generate pentose phosphates. Two enzymes in the oxidative branch generate NADPH: glucose-6-phosphate dehydrogenase (G6PDH) and 6-phosphogluconate dehydrogenase (6PGDH). G6PDH is activated upon dimerization, and catalyzes the rate-limiting step of the PPP (Cohen and Rosemeyer, 1969). G6PDH requires NADP+ for activation and is inhibited by high NADPH. The NADPH generated by the PPP is primarily used for reductive biosynthesis, with isotopic flux tracing showing that up to 80% of NADPH production was compromised by knockdown of G6PDH in proliferating cells (Fan et al., 2014). The PPP also drives NADPH production in the ER through hexose-6-phosphate dehydrogenase (H6PD) (Clarke and Mason, 2003). H6PD catalyzes the same conversion as G6PDH, but exhibits broader substrate specificity. The NADPH produced by H6PD is required for interconversion of cortisol and cortisone by 11β-hydroxysteroid dehydrogenase (Atanasov et al., 2004). ER lumenal NADPH is probably uncoupled from oxidative protein folding, because there are no known glutathione or thioredoxin reductases in the ER (Piccirella et al., 2006).

### One-Carbon Metabolism

One-carbon (1C) metabolism includes the linked folate and methionine cycles. Folate supports 1C metabolic oxidations and reductions through interconnected mitochondrial and cytosolic half-cycles (Ducker and Rabinowitz, 2017). 1C units are primarily derived from serine and glycine, and are presumably generated independently in the mitochondria and cytosol because there is no known transfer of 1C-bound folates across membranes (Anderson et al., 2011). Because mammals do not synthesize folate *de novo*, dietary folate is required for 1C metabolism. Dietary folate is reduced to tetrahydrofolate (THF), which is a universal 1C acceptor that can hold 1C units in three interconvertible oxidation states. The three oxidation states determine which biosynthetic process the 1C unit can be used for—purine synthesis, thymidine synthesis, and homocysteine remethylation. In the mitochondria, THF is oxidized through a series of NADP+-dependent reactions to generate formate, which is exported to the cytosol where it is reduced by NADPH-dependent reactions to regenerate methionine, or oxidized—again relying on NADP+—for purine synthesis.

1C metabolism is regulated by the ratio of NADP+ to NADPH. When the NADP+:NADPH ratio is high, the mitochondria favor serine oxidation to fuel 1C metabolism and restore NADPH levels through the activity of methyl-tetrahydrofolate dehydrogenase 2 and aldehyde dehydrogenase 1 family member L2, which are reportedly sufficient to maintain mitochondrial NADPH (Fan et al., 2014; Lewis et al., 2014). Moreover, high NADP+ inhibits cytosolic folate metabolism by decreasing dihydrofolate reductase (DHFR) activity, presumably to prevent NADPH consumption (Chen et al., 2019). This regulation by the NADP+:NADPH ratio in both compartments might make 1C metabolism responsive to NADPH generated by PPP and the TCA cycle. The extent to which NADPH production by 1C metabolism is affected by changes in metabolic flux have not been fully characterized. However, recent evidence indicates that 1C metabolism supports NADH production when the TCA cycle is inhibited (Yang et al., 2020). This linkage implies that the same may be true for NADPH—that diminished TCA cycle activity might stimulate NADPH production by 1C metabolism—though the enzymes in 1C metabolism that produce NADH and NADPH rely on the same folate derivatives, and it remains unclear whether the cell would prioritize reducing NAD+ over NADP+.

### The Tricarboxylic Acid Cycle

While the TCA cycle is commonly thought of as an NADH and FADH_2_ producing pathway, it also generates NADPH. Three TCA isozymes—malic enzyme 1 (ME1), and isocitrate dehydrogenase 1 and 2 (IDH1/2)—produce NADPH (Rydström, 2006). ME1 and IDH1 produce NADPH in the cytosol, meaning their substrates, malate and isocitrate, must be exported from the mitochondria. Conversely, IDH2 generates NADPH in the mitochondrial matrix to support mitochondrial redox homeostasis. Both ME and IDH have NAD+ dependent counterparts that only reside in the mitochondria and generate the NADH needed for electron transport chain activity. ME1 and IDH1 are able to compensate for loss of G6PDH (Chen et al., 2019), meaning that the cytosolic pools of NADPH are, to some extent, interchangeable. It is less clear how ME1 and IDH1 activity affects the activity of IDH2, and vice-versa and whether these two enzymes compete for the same pools of (iso) citrate.

## Pathways Linking Reduced Nicotinamide Adenine Dinucleotide Phosphate to Endoplasmic Reticulum Function

**FIGURE 3:**
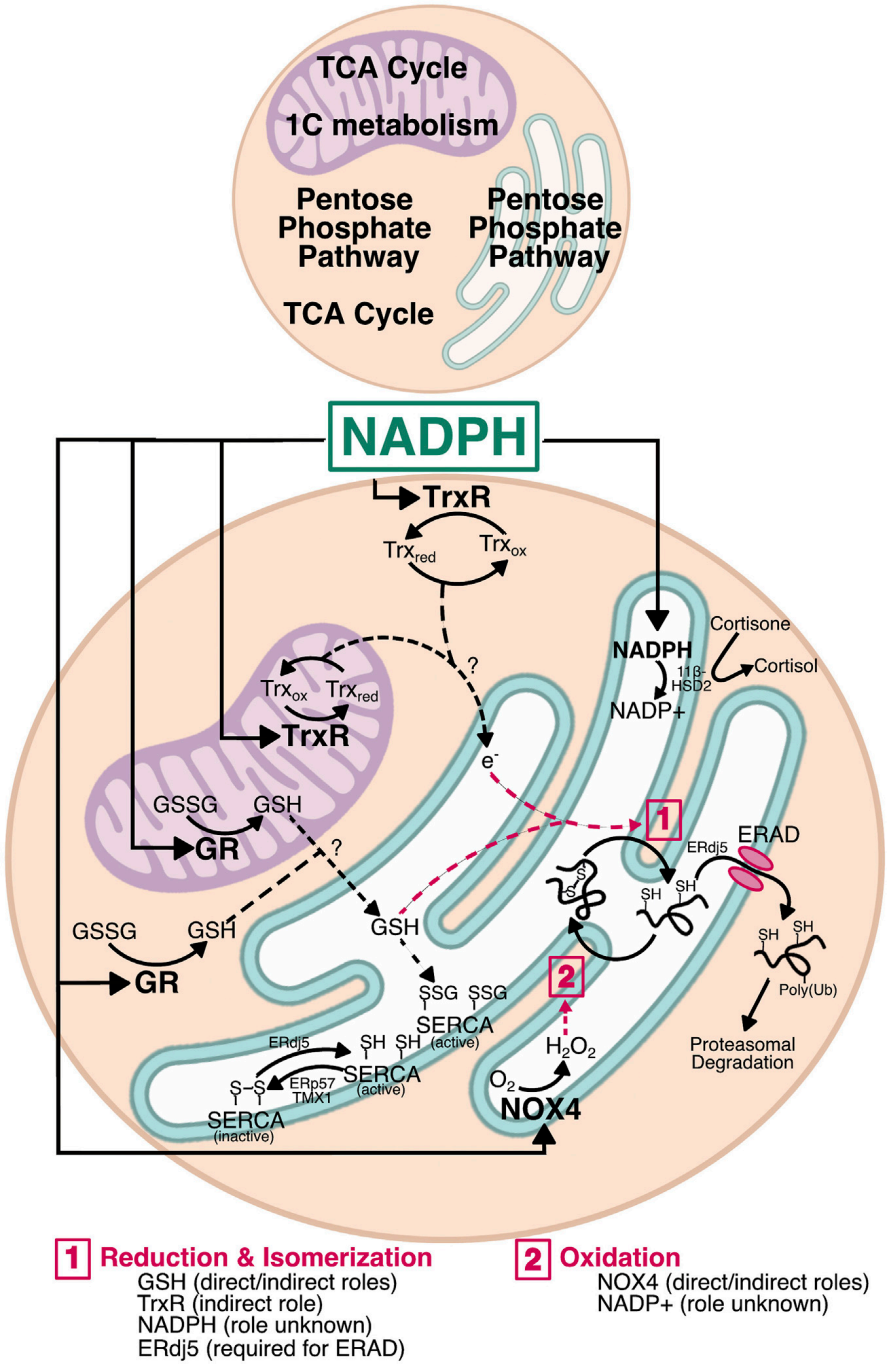
Mechanisms by which NADPH could affect ER function. NADPH is a major factor in redox homeostasis. In both the cytosol and mitochondria, NADPH is used by glutathione reductase (GR) and thioredoxin reductase (TrxR). Generation of GSH by GR in either compartment could move into the ER and alter the oxidizing environment by interacting with PDI family members for disulfide reduction, whether during isomerization or for retrotranslocation. Similarly, TrxR has the potential to transfer electrons across the ER membrane—by a currently unknown mechanism—for reduction of non-native disulfides during isomerization. Reduction by ERdj5 is also required for protein retrotranslocation during ERAD. GSH and electrons entering *via* the Trx pathway may also influence ER calcium by regulating SERCA. SERCA is activated by reduction (ERdj5) and inactivated by oxidation (ERp57 and TMX1), and is also activated by S-glutathionylation, providing a direct role for glutathione in calcium homeostasis. Finally, NADPH oxidase 4 (NOX4) uses cytosolic NADPH to generate H_2_O_2_ in the ER lumen. This H_2_O_2_ could be used by ER-resident PRDX4 and GPx7/8 during PDI reoxidation as shown in Figure 1. The H_2_O_2_ produced by NOX4 also has the potential to regulate SERCA redox and calcium homeostasis. Though the NADPH pool in the ER lumen appears uncoupled from the protein folding machinery, it nonetheless plays a role in overall redox and regulates steroid activation by 11β-hydroxysteroid dehydrogenase (11β-HSD). Whether there are reductases that use NADPH in the ER lumen remains unknown, and thus a role for NADPH in protein folding cannot be fully excluded.

It is becoming clear that the ER lumen is responsive to changes in NADPH levels outside the ER. For example, cytosolic NADPH plays a role in reduction of non-native disulfides that form in the ER (Poet et al., 2017). NADPH is also implicated in regulating N-linked glycosylation efficiency, since both the ER glycosylation machinery and the proteins being glycosylated are redox sensitive (Van Lith et al., 2021). Similarly, we recently showed that NADPH generated by the TCA cycle leads to a hypo-oxidizing ER and, surprisingly, increased sensitivity to ER stress (Gansemer et al., 2020). In this section, we will describe three major pathways by which NADPH levels can be transmitted to the ER lumen (Figure 3). As will be discussed, glutathione is one possible candidate because it relies on NADPH for reduction, which is thought to occur in the mitochondria and cytosol, but not the ER (Lu, 2013; Ribas et al., 2014; Couto et al., 2016), and a potential mechanism for the entry of reduced glutathione into the ER has been characterized in yeast (Ponsero et al., 2017). The thioredoxin pathway, which also utilizes NADPH, is another candidate. Although thioredoxin is not itself imported into the ER (being an 11.6 kDa protein rather than a small tripeptide like glutathione), the pathway has been shown to influence ER lumenal redox potential, likely through the conveyance of reducing equivalents through an as-yet unknown ER-resident transmembrane protein (Poet et al., 2017). Finally, NADPH also can influence lumenal redox—and potentially protein folding—through production of peroxides by transmembrane NADPH oxidases (NOX).

### Glutathione Redox

Glutathione is a tripeptide consisting of glycine, cysteine, and glutamate, with the cysteine linked to the carboxyl group of the glutamate side chain by a γ-peptidyl bond; it is synthesized in the cytosol by glutathione synthase (Lu, 2013). The sulfhydryl group of the cysteine residue can form disulfide bonds with other reactive cysteines, including in another glutathione molecule to form oxidized glutathione (also called glutathione disulfide, GSSG). Reduced glutathione (GSH) is negatively charged at physiological pH, which contributes to the requirement for facilitated transport across membranes. Additionally, the differences in the relative ratio of GSH:GSSG between subcellular compartments argues against passive *trans*-ER movement of both GSSG and GSH; the cytosol and mitochondrial matrix have high GSH:GSSG ratios of approximately 100:1, whereas the ER has a substantially lower ratio of closer to 3:1 (Forman et al., 2009; Appenzeller-Herzog, 2011; Ribas et al., 2014). If glutathione could move passively between compartments, maintaining these ratios would be impossible. Despite the requirement for facilitated transport, glutathione movement between subcellular compartments is not well understood. GSH was thought to be exchanged between the mitochondrial intermembrane space and matrix by the 2-oxoglutarate and dicarboxylate carriers (Chen and Lash, 1998; Chen et al., 2000), though this has since been challenged (Booty et al., 2015). Recently, the mitochondrial solute carrier SLC25A39 was identified as a regulator of mitochondrial GSH import (Wang et al., 2021), though it remains unclear how mitochondrial GSH is exported. Transport into and out of the ER is even less understood, with the only identified candidate for GSH transport being the Sec61 translocon in yeast (Ponsero et al., 2017). There is evidence of glutathione nanodomains at ER-mitochondrial contact sites (Booth et al., 2021), and thus it is plausible that GSH—or GSSG—could be directly transferred from the mitochondria to the ER at those sites.

Glutathione levels are not only regulated by transport, but also by redox cycling. GSH is oxidized to GSSG by glutathione peroxidase (GPx) in the process of peroxide detoxification, and GSSG is reduced to GSH by glutathione reductase (GR) to maintain reductive power. GPx enzymes use either a catalytic cysteine or selenocysteine to mediate this reaction. To date, eight mammalian GPx isoforms (GPx1-8) have been characterized (Brigelius-Flohé and Maiorino, 2013). The GPx family has been extensively reviewed elsewhere (Brigelius-Flohé and Maiorino, 2013). Briefly, GPx1 is widely expressed, is required for mitigating oxidative stress, and has been identified in the cytosol and mitochondria (Esworthy et al., 1997; Lubos et al., 2011). GPx4 was the first monomeric GPx identified, and is also widely expressed. It is localized to the cytosol and mitochondria, and reacts with GSH and lipid peroxides present in membranes or lipoproteins (Thomas et al., 1990). GPx4 also has high affinity for protein thiols, and can thus act as a thiol peroxidase when GSH is limiting. GPx4 is particularly highly expressed in the liver, lungs, kidneys, and brain, and loss of GPx4 increases apoptosis and lipid peroxidation (Brigelius-Flohé and Maiorino, 2013). GPx2, 3, 5, and 6 have more narrow expression. GPx7 and GPx8 are/ER-localized, with GPx7 being the first GPx identified with a cysteine instead of a selenocysteine (Utomo et al., 2004). GPx7 is localized to the ER lumen, where it uses H_2_O_2_ generated by ERO1 and NOX4 to re-oxidize PDI during disulfide formation (Wang et al., 2014). GPx8 is similar to GPx7, but is instead a single-pass ER membrane protein with a lumenal C-terminus. Less is known about the function of GPx8, though it also participates in PDI reoxidation (Nguyen et al., 2011; Kanemura et al., 2020) and prevents H_2_O_2_ leakage from the ER (Ramming et al., 2014).

Changes in GPx expression and activity are closely tied to both metabolism and ER function. Loss of GPx1 induces ER stress (Geraghty et al., 2016; Gansemer et al., 2020), and its inhibition makes disulfide-bonded ER client proteins more sensitive to reduction (Gansemer et al., 2020). Similarly, GPx8 suppression was recently shown to enhance ER stress in the liver (Lee et al., 2021). That these manipulations cause ER stress—or, more specifically, induce the UPR—and alter the ER redox environment implies that they do so by affecting the folding, maturation, and secretion of proteins transiting through the ER, but such an effect has not yet been directly demonstrated. If impairing GPx expression or activity elicits or exacerbates ER stress, then overexpressing it should attenuate ER stress. While this prediction has not yet been tested, it is worth noting that overexpression of GPx1 augments pancreatic beta-cell insulin production (Harmon et al., 2009). This effect might arise at least in part from enhanced insulin processing in the ER, because insulin structure and stability rely on formation of two interchain disulfides and one intrachain disulfide (Van Lierop et al., 2017). However, it is also possible that this effect is due to the generally salubrious effects of protection from oxidative stress that would be expected from augmenting a glutathione peroxidase; disentangling the role of glutathione in detoxifying peroxides from its contribution to ER oxidative folding remains a challenge.

The second half of the glutathione redox cycle is catalyzed by GR, an NADPH-dependent flavoprotein (Couto et al., 2016). The NADPH and FAD binding domains permit transfer of electrons from NADPH to FAD. A pair of reactive cysteines form an intramolecular disulfide bond that then shuttles electrons from FADH_2_ to GSSG, reducing GSSG to two GSH molecules and re-oxidizing themselves in the process. GR activity has been detected in the mitochondria and cytosol, and the protein contains an N-terminal mitochondrial targeting sequence (Kelner and Montoya, 2000). In yeast, this dual localization is achieved by translation from two start sites (Outten and Culotta, 2004), though the mechanism of dual localization of the mammalian GR has not been determined. In any case, its presence in both compartments comports with the presence of NADPH-generating pathways and the need to provide reducing equivalents in both places. The cytosol needs to be reducing to prevent protein disulfide bonding and aggregation, and to prevent or rectify oxidative damage. Similarly, the mitochondria have a high demand for reducing equivalents due to ROS production by oxidative phosphorylation. The mitochondria thus support detoxification of ROS produced during oxidative phosphorylation by generating NADPH through IDH2 and one-carbon metabolism, effectively ensuring that mitochondrial GR has sufficient NADPH to regenerate GSH and maintain a reducing environment.

Unlike the cytosol and mitochondrial matrix, the ER lumen demands more oxidizing conditions and is clearly adversely affected when its oxidation potential is insufficient. The apparent lack of GR activity in the ER suggests that the redox state of ER lumenal glutathione is instead controlled by import of GSH that is generated in the cytosol or mitochondria. In this way, high metabolic activity and its consequent increased NADPH production could affect ER function through GR activity, even though GR itself is not in the ER. The idea that NADPH and GR activity could affect the ER is indirectly supported by evidence that stimulating TCA activity increases NADPH and GSH, and induces ER stress. The converse is also true, as blocking TCA entry of either carbohydrates or fats decreases NADPH and GSH, makes the ER more oxidizing, and diminishes ER stress—an effect that can be phenocopied by inhibiting GR (Gansemer et al., 2020).

### Thioredoxin Reductase

The thioredoxin reductases (TrxR) are structurally and functionally similar to GR. TrxRs are also NADPH-consuming flavoproteins that exhibit cytosolic (TrxR1) and mitochondrial (TrxR2) localization in mammals. The TrxRs differ from GR by having a catalytic selenocysteine redox-active site at the C-terminus in addition to the standard CXXC domain (Lu and Holmgren, 2014). This selenocysteine is required for TrxR activity. The Trx system is primarily responsible for reducing disulfides in the cytosol (Lu and Holmgren, 2014). TrxR binds FAD, allowing it to accept electrons from NADPH. The resultant FADH then reduces an intramolecular disulfide within TrxR, and the electrons are eventually transferred to the small redox-active protein thioredoxin. Reduced thioredoxin is capable of interacting with and reducing disulfide bonded thiols. Reducing disulfides in the cytosol prevents protein aggregation and promotes proper protein localization. There is considerable overlap among the glutathione and thioredoxin pathways, and loss of either pathway alone is generally well-tolerated (Miller and Schmidt, 2019).

The effects of the Trx system extend to the ER, with cytosolic TrxR1 surprisingly being required for reducing non-native disulfides during oxidative folding in the ER (Poet et al., 2017). Further analysis in an *in vitro* reconstituted system determined that an as-yet unidentified ER membrane protein or proteins are required for Trx and TrxR1 to exert their effects on ER disulfide reduction (Cao et al., 2020; Van Lith et al., 2021). Its proteinaceous nature was determined by showing that the ability of Trx to mediate the reduction of lumenal components was protease-sensitive. Because such a protein must facilitate the net transfer of reducing equivalents across the ER membrane, the most likely mechanism is a protein or protein complex for which reduction by Trx in the cytosol elicits a conformational change that allows the electrons to be shuttled intramolecularly to a lumenal disulfide. The oxidation status of such a protein should change in a Trx-dependent manner, and loss of that protein should make the ER more oxidizing. One potential candidate is LMF1, which is an ER-resident transmembrane protein that facilitates the maturation and secretion of lipoprotein lipase. LMF1 deletion indeed rendered the ER more oxidizing (and compromised LPL secretion) (Roberts et al., 2018). LMF1 contains three cytosolically disposed cysteines, two of which are in a juxtamembrane region and so might be best positioned to transmit changes in oxidation status across the membrane—though such a role is currently purely speculative. Nonetheless, this evidence further implicates NADPH—and by extension metabolic flux—in ER homeostasis. While reduction of non-native disulfides is essential, the possibility that excess NADPH production causes reduction of native disulfides cannot be excluded.

The Trx system is also linked to changes in metabolism and the progression of metabolic disease, which could also be directly or indirectly related to ER function. Obese individuals (Heinonen et al., 2015) and genetically obese (ob/ob) mice exhibit elevated TrxR activity in the liver and adipose tissue (Rojanathammanee et al., 2014). In contrast, dietary models of obesity led to TrxR2 upregulation in muscle (Fisher-Wellman et al., 2013), but decreased TrxR and Trx in the liver (Qin H. et al., 2014). To the extent that the Trx system impinges on ER function, ER homeostasis is likely to be sensitive to metabolic fluxes through this pathway. The apparent discrepancy between the dietary and genetic models, and how overnutrition would then affect ER homeostasis through the Trx system, might be due to the different ways that the two models induce obesity. Animals fed high fat or western diets consume more calories, but do not necessarily exhibit hyperphagia, whereas ob/ob animals consume more calories because of hyperphagia. Since the simple act of feeding induces ER stress independent of dietary fat content, at least in the liver (Gomez and Rutkowski, 2016), it is possible that the consequences of obesity on ER function depend on whether or not the stimulus is experienced as accelerated feeding and fasting cycles, as in ob/ob mice, or as more calorically potent episodes of feeding with relatively normal frequency.

### NADPH Oxidases

The NADPH oxidases (NOX) are six-pass transmembrane proteins that transfer electrons from NADPH across a membrane to molecular oxygen, ultimately generating ROS in the form of superoxide or H_2_O_2_ (Panday et al., 2015). All NOX enzymes have C-terminal cytosolic NADPH binding domains, and an FAD domain between the NADPH binding domain and the first transmembrane domain. NOX enzymes are functionally similar to GR, employing FAD binding to transfer electrons from NADPH. However, NOX enzymes contain heme-binding histidine residues within the third and fifth transmembrane domains that can accept electrons before transferring them to oxygen on the opposite, exofacial or lumenal side of the membrane. There are five NOX enzymes (NOX1-5) and two closely related dual oxidases (DUOX1/2), which contain an NADPH oxidase domain and a peroxidase domain. The NOX enzymes are functionally regulated by protein-protein interactions, with the interaction between NOX and p22phox being conserved for NOX1-4 (Brandes et al., 2014). NOX5 and DUOX1/2 are regulated by calcium instead of protein-protein interactions. The membrane topology of NOX proteins also influences their activity. Both the N- and C-termini are thought to be cytosolic, which is essential because of the reliance of these enzymes on cytosolic components such as p47phox, p40phox, p67phox, and Rac for activation (Dusi et al., 1996). This topology favors the use of cytosolic NADPH by NOX enzymes. NOX1-3 and DOUX1/2 are localized to the plasma membrane, whereas NOX4 has been identified in the ER and mitochondrial membranes, and in the nucleus (Kuroda et al., 2005; Laurindo et al., 2014; Shanmugasundaram et al., 2017). Little is known about NOX5, but it is reportedly localized to organellar membranes in the absence of stimulation and traffics to the plasma membrane in the presence of phosphatidylinositol 4,5-biphosphate (Kawahara and Lambeth, 2008).

As transmembrane proteins, NOX enzymes are synthesized and processed, and heme is incorporated, at the ER (Wallach and Segal, 1997) (Isogai et al., 1995). The UPR reportedly upregulates NOX4, which is unsurprising because NOX enzymes are commonly regulated at the transcriptional level (Laurindo et al., 2014; Bettaieb et al., 2015). This upregulation implies that there is a functional benefit to the ER in inducing NOX4 activity. It is plausible that the UPR activates NOX4 as a means to promote protein oxidation by generating H_2_O_2_ for PDI reoxidation by PRDX4 and GPx7/8 (Figure 1). Such a role would be consistent with the fact that the UPR also upregulates ERO1α expression, suggesting that augmenting ER oxidative folding is part of the UPR adaptive program. Alternatively, it is possible that, in the face of unremitting stress, such augmentation might be cytotoxic due to the ROS burden (Li et al., 2009; Li G. et al., 2010). Additionally, NOX4 plays a role in sustaining UPR activity by activating the PERK pathway (Sciarretta et al., 2013; Sun et al., 2021). Sustained PERK activation could explain why NOX4 is implicated in liver and adipose tissue inflammation, though this has not been directly tested.

The role of NOX4 in obesity and overnutrition remains controversial. The consensus is that increased NOX4 contributes to or exacerbates obesity and obesity-related phenotypes, which was derived from observed increases in NOX4 in adipose tissue, muscle, and liver during overnutrition. However, studies have found that deleting NOX4 can either exacerbate or attenuate obesity and obesity-related phenotypes, depending on the model system: Liver-specific deletion/inhibition of NOX4 decreased inflammation and fibrosis (Jiang et al., 2012; Lan et al., 2015). Similarly, adipocyte-specific deletion attenuated inflammation and delayed the onset of insulin resistance in diet-induced obesity. On the other hand, global deletion predisposed mice to diet-induced obesity and hepatic lipid accumulation (Li et al., 2012).

NOX4 also appears to affect cell proliferation. NOX4 deletion accelerated hepatocyte proliferation after partial hepatectomy (Herranz-Itúrbide et al., 2021)—which is an essential component of the response—but also accelerated proliferation and migration in hepatocellular carcinoma (Crosas-Molist et al., 2014), suggesting that inhibiting NOX4 could exacerbate the progression of liver disease associated with obesity. Similarly, NOX4 regulates the balance between adipocyte differentiation and proliferation, with NOX4 deficiency promoting proliferation and decreasing insulin-induced differentiation (Schröder et al., 2009). Whether these effects arise from an altered ER lumenal redox environment has not been tested.

## Pathways by Which Altered Endoplasmic Reticulum Redox can Affect Endoplasmic Reticulum Homeostasis

By generating NADPH and its associated reducing power, nutrient catabolism can be expected to set in motion pathways that ultimately influence the ER redox state: production of GSH which can be directly imported into the ER; reduction of thioredoxin, which can lead to reduction of lumenal proteins through a transmembrane intermediate; and activation of NOX4 to generate lumenal H_2_O_2_, which would in contrast favor oxidation in the lumen by supplying H_2_O_2_ for PRDX4 and GPx7/8. Our data show that suppressing production of NADPH leaves the ER more oxidizing (or, to be more precise, more resistant to reduction) and, consequently, more resistant to an exogenous ER stressor (Gansemer et al., 2020). In broad terms, this finding suggests that dysregulation of metabolic pathways, as occurs in obesity, is likely to affect how well the ER can withstand challenges. But, even more intriguingly, it raises the possibility that ER redox and protein processing capacity fluctuate in tandem with feeding and fasting cycles. In the simplest scenario, the ER would be more oxidizing during fasting conditions and more reducing upon nutrient intake and catabolism; thus, disulfides on ER client proteins would form more readily during fasting, and be reduced or isomerized more readily during feeding. It might be expected, then, that nutrient intake would benefit the maturation of ER client proteins with nonconsecutive disulfide bonds such as antithrombin or the LDL receptor (Chandrasekhar et al., 2016; Kadokura et al., 2020) and perhaps compromise or delay the maturation of proteins with consecutive disulfides such as albumin. However, these two straightforward predictions—that nutrient intake will make the ER less oxidizing and that disulfide bonds will then form less readily—have not to our knowledge been tested. Moreover, it is conceivable that whether this relationship holds true or not will depend on how metabolically active a given cell type is, with cell types such as hepatocytes, adipocytes, and myocytes, which are highly metabolically active, being more sensitive to nutrient fluxes than less active cell types like fibroblasts (Frayn et al., 2006).

Beyond having potentially direct effects on the oxidation of ER client proteins, ER redox can also impact homeostasis in the organelle indirectly. This effect arises because the proteinaceous machinery within the ER has evolved to work optimally within the highly oxidizing ER lumen, and contains reactive thiols that allow their activity to be modulated when ER redox is perturbed. Three pathways in particular—ERAD, calcium storage, and UPR signaling—have received particular attention.

### Endoplasmic Reticulum-Associated Degradation

ERAD involves the recognition of terminally misfolded ER client proteins and their dislocation through a channel into the cytosol for destruction. It is thought that substrate reduction is a prerequisite for degradation, and ERAD substrates have long been known to associate with thiol isomerases including ERp90, ERp57, and PDI (Gillece et al., 1999; Tsai et al., 2001; Schelhaas et al., 2007). More recently, the thiol isomerase ERdj5 has emerged as an important reductase for misfolded proteins. ERdj5 interacts with the ER chaperone BiP through its J-domain and with ERAD client proteins and the ER mannosidase EDEM through its thioredoxin domains (Ushioda et al., 2008; Hagiwara et al., 2011). EDEM trims N-glycan structures on misfolded proteins, allowing them to interact with lectins such as OS9 that shuttle such proteins to the ERAD machinery (Määttänen et al., 2010). Thus, it is thought that ERdj5 recognizes misfolded substrates by virtue of their association with EDEM, reduces them, and then transfers them to the ERAD machinery. Further, ERdj5 can also recognize and reduce non-glycoproteins (Ushioda et al., 2013) and EDEM itself is also capable of recognizing misfolded proteins through thiol-mediated interactions apart from carbohydrate binding (Lamriben et al., 2018). ERdj5 was shown to be essential for correct disulfide bond formation on the LDL receptor (Oka et al., 2013). The apparent redox equilibrium constant of ERdj5 is approximately 100-fold higher than that of the ER at large (Ushioda et al., 2008); in other words, under the normal redox conditions of the ER lumen, ERdj5 exists almost exclusively in the oxidized form and acts only as a reductase. Reduction of ERdj5 by some other component—as yet unknown—would drive the reductive activity of ERdj5 toward its substrate so that it could return to its more stable oxidized form. This unique feature of ERdj5 is likely a consequence of the C-X-P-C motif in its thioredoxin domains (Roos et al., 2007). Whether ERdj5 activity is altered by nutrient fluxes has not been tested. EDEM also forms a mixed disulfide bond with the thiol isomerase ERp46, which was required for substrate mannose trimming (Yu et al., 2018).

### Calcium Storage

Another major role of the ER is calcium storage and regulated release. The Sarco/Endoplasmic calcium ATPase (SERCA) proteins are responsible for importing calcium into the ER from the cytosol against its steep concentration gradient. The IP3 receptor (IP3R) and ryanodine receptor (RyR) control its regulated release. ER calcium depletion has long been recognized as an ER stressor (Price et al., 1992; Li et al., 1993). Many ER chaperones bind calcium with low affinity, and function optimally in the high calcium conditions of the ER lumen. More specifically, it was recently shown that calcium increases the affinity of BiP for ADP, so that high calcium promotes substrate retention by BiP while low calcium promotes release of (potentially unfolded) substrate (Preissler et al., 2020). The activities of SERCA, IP3R, and RyR have all been shown to be redox sensitive; these regulatory steps are well-reviewed in (Appenzeller-Herzog and Simmen, 2016; Chernorudskiy and Zito, 2017). Redox regulation of SERCA is the best-studied of these.

SERCA can be redox-regulated from both the cytosolic and lumenal sides of the ER membrane. In the cytosol, reducing conditions favor SERCA glutathionylation and an increase in activity (Adachi et al., 2004), while oxidizing conditions cause sulfonation and impair SERCA activity (Qin et al., 2013; Qin F. et al., 2014). In the lumen, SERCA contains a regulatory disulfide that can be oxidized by ERp57, decreasing SERCA activity (Tong et al., 2010). This process is regulated in the ER by calreticulin under conditions of high calcium, comprising a negative feedback loop to prevent overfilling of calcium. SERCA can also be inactivated by the thioredoxin-related transmembrane protein TMX1 (Raturi et al., 2016). The disulfide can be reduced by ERdj5 (Ushioda et al., 2016) or selenoprotein N (SEPN1/SELENON) (Marino et al., 2015). SELENON forms disulfide-linked homo-oligomers that dissociate when ER calcium is depleted, and interact *via* a disulfide bridge with SERCA2 (Chernorudskiy et al., 2020). Thus, just as elevated ER calcium acts through a redox relay to inhibit SERCA, low ER calcium activates it. SELENON also retards the reoxidation of PDI when ERO1 is absent (Pozzer et al., 2019), hinting that SELENON plays a larger role in the maintenance of ER redox. SERCA activity is also maintained by the ER lectin chaperone calnexin, which maintains a basal level of oxidation of SERCA that is required for its activity. When this regulation is compromised, the ER and mitochondria become much more closely apposed, and mitochondrial catabolic activity is diminished (Gutiérrez et al., 2020). How calnexin accomplishes this regulation is unclear, though NOX4 might be involved (Prior et al., 2016).

SERCA expression and activity showed a circadian oscillation in rat liver when food intake was restricted (Báez-Ruiz et al., 2013), hinting at possible metabolic regulation. Likewise, a luciferase reporter that is released from the ER and secreted upon ER calcium depletion (Henderson et al., 2014) pointed to possible disruptions to ER calcium homeostasis in the livers of rats fed high fat diets (Wires et al., 2017). Consistent with this idea, more recently obesity was found to inhibit SERCA in the liver; this effect was attributed to alterations in cellular lipid composition (Fu et al., 2011) SERCA activity can also be regulated by metabolites like glucose-6-phosphate (Cole et al., 2012) and phosphoenolpyruvate (Ho et al., 2015). Yet by and large, the molecular mechanisms linking metabolism to SERCA activity, and whether they are mediated through the ER redox pathways described above, are not clear.

### Unfolded Protein Response Signaling

In addition to the redox effects of NADPH affecting ER protein folding, they might also affect the sensitivity of the ER-resident UPR stress sensors, in effect uncoupling UPR signaling from the ER folding environment. ATF6 contains intrachain disulfide bonds that must be reduced by the PDI family member PDIA5 during ER stress in order for ATF6 to be activated. However, an interchain disulfide must then form; both the reduction and oxidation steps are required for ATF6 activation (Nadanaka et al., 2007; Higa et al., 2014; Oka et al., 2022). In the absence of ER stress, the status of the lumenal cysteines in both IRE1 and PERK is unknown. During ER stress, interchain disulfide formation facilitates IRE1 activation, and reduction by another PDI family member, PDIA6, is required for deactivation (Li H. et al., 2010; Eletto et al., 2014b). IRE1 is also susceptible to the oxidative modifications nitrosylation and sulfenylation, which can disrupt IRE1 kinase activity and preferentially activate the antioxidant response (Yang et al., 2015; Hourihan et al., 2016). Little is known about the role of the lumenal cysteines in PERK activation, though dimerization and reduction contribute to activation and inactivation, respectively (Eletto et al., 2014a; Kranz et al., 2017). In theory, a hyperoxidizing ER could preferentially sustain IRE1 and PERK activity, while preventing activation of ATF6. Therefore, changes in redox could impact sensitivity to other ER stressors. For example, an increase in oxidizing equivalents could induce IRE1 and PERK independently of the folding status of ER client proteins, which might then diminish the ability of IRE1 and PERK to sense bona fide ER stress. Whether such modulation of sensor activation would benefit the ER or not remains less clear.

## Conclusion and Perspectives

This review aimed to highlight the mechanisms by which NADPH could act as a bridge between nutrient availability and ER protein processing. In simplistic terms, we propose that increased nutrient catabolism drives NADPH production, which in turn renders the ER hypo-oxidizing, likely through the glutathione and possibly thioredoxin pathways. Because NADPH regulates redox homeostasis across subcellular compartments, and the various pools appear to interact, it is difficult to fully disentangle the degree of influence each pool has on ER function. Furthermore, identifying the major pathway linking NADPH and ER function is complicated by the fact that NADPH regulates three major redox pathways: glutathione, thioredoxin, and NOX enzymes, each of which interacts—either directly or indirectly—with protein folding machinery in the ER. A major question moving forward is the degree to which these three pathways act redundantly versus uniquely on the ER oxidation capacity. While glutathione was once thought to be the major driver of ER oxidative folding, more recently a functional role for GSSG in the ER lumen has fallen out of favor, as impairment of glutathione synthesis in yeast (Frand and Kaiser, 1998) and ER glutathione depletion in mammalian cells (Tsunoda et al., 2014) failed to compromise ER protein oxidation. However, the observation that inhibition or knockdown of GR diminishes ER stress and that knockdown or inhibition of GPx1 causes it (Gansemer et al., 2020) argues that, at least in hepatocytes, GSSG plays some functional role within the ER lumen. Whether this role is direct or indirect awaits measurement of the GSSG concentration specifically in the ER lumen when GR is inhibited, as well as its effect on the oxidation of nascent ER client proteins. Likewise, whether similar protection from ER stress can be phenocopied by inhibition of TrxR or NOX4 remains to be determined.

There is also the question of whether ER function is benefited by more oxidizing or more reducing conditions—or whether this depends on cell type—and how a hyper- or hypo-oxidizing ER is interpreted by the cell (or not) as ER stress. With a role for GSSG in protein oxidation being currently disfavored, it is now thought that a primary role of glutathione is to provide reducing equivalents for the breaking of non-native disulfides (Delaunay-Moisan et al., 2017; Ellgaard et al., 2018). One purpose of this reduction would be to maintain PDI in the reduced state, which is the configuration required for it to isomerize disulfide bonds. Reduction can also be in the service of ER-associated degradation, which requires client proteins to be reduced prior to their dislocation from the ER lumen to the cytosol prior to proteasomal disposal (Ushioda et al., 2008; Hagiwara and Nagata, 2012). For these reasons, it would seem that hyperoxidizing conditions in the ER could be more detrimental to ER function that hypooxidizing conditions, because the former could potentially trap proteins in non-native disulfide bonded forms, preventing their proper folding or degradation. Yet despite the appeal of this logic, reducing agents remain much more potent ER stress-inducing agents (or, more precisely, UPR activating agents) than do treatments that oxidize the ER. This discrepancy raises the question of whether a hypo-oxidizing ER is therefore a more profound disruptor of ER function, or if UPR activation simply responds more readily to a hypo-oxidizing ER than to a hyper-oxidizing one, even if both perturb protein folding and processing. Or are the UPR sensors themselves redox regulated in a way that dissociates their activation from true ER folding conditions during redox alterations? Or are all of these possibilities true at some level, depending on the cell type and nutritional conditions? These questions require the analysis of a broad range of ER client proteins, in an array of different cell types, and how their biogenesis, secretion, and degradation, and UPR activation are influenced by the cellular redox state.

There is also the question of how redox signals are exchanged across membranes. Of particular interest are IDH1 and IDH2, which catalyze the same NADPH-generating reaction but in the cytosol and mitochondria, respectively. When TCA cycle flux is high, is IDH2 the primary NADPH generator since it is housed within the mitochondrial matrix, or is citrate or isocitrate exported from the mitochondria to feed IDH1 activity? If IDH2 is active while IDH1 is inactive, is the GSH that is generated in the mitochondria nonetheless exported to the cytosol, such that it will be elevated in both compartments? And, likewise, if IDH1 is active, is GSH then imported into mitochondria, or is the elevation of GSH restricted to the cytosol? And does the location of GSH elevation affect its ability to influence the ER lumenal redox environment? One might imagine that cytosolic elevation of GSH would more readily affect ER lumenal redox than would mitochondrial GSH elevation because of the additional barrier posed by the mitochondrial membranes. However, if glutathione can be readily exchanged between mitochondria and the ER at mitochondrial-ER contact sites, then, paradoxically, IDH2 activity might more readily influence ER redox than IDH1. Alternatively, perhaps changes to glutathione in redox are readily transmitted across all three compartments (mitochondria, bulk cytosol, and ER) by mass action. In this case, it would be expected that the promotion of ER oxidative folding in the ER and the defense against reactive oxygen species in the cytosol would be fundamentally competing processes. The availability of compartment-specific biosensors for NADPH/NADP+ and GSSG/GSH (Gutscher et al., 2008; Tao et al., 2017; Sallin et al., 2018) will be indispensable in sorting among these possibilities.

Finally, why has evolution selected for a UPR that responds to metabolites produced by catabolism? One intriguing possibility is that the ER stress elicited by a single feeding event is by itself not sufficiently strong or long-lasting to cause significant real disruption to ER function, but is merely robust enough to activate the UPR in anticipation of the increased protein biogenesis load caused by mTOR activation during the postprandial period. In this view, NADPH and, by extension, GSH, are essentially sentinel molecules that warn the ER machinery of impending stress. If this were the case, then impairment of either NADPH production or glutathione reduction, even though it protects the ER against stresses applied contemporaneously (Gansemer et al., 2020), might actually sensitize the ER to stresses, such as increased protein biogenesis, that occur later. Examining in animals whether loss of enzymes such as IDH1/2 or GR is protective or sensitizing against pharmacological ER stresses on one hand, and overnutrition on the other, are needed to test this idea.

Delimited by its own membrane enclosure, it is easy to view the ER as detached from the goings-on of the cell around it. But because metabolism and redox are the fundamental chemistries of life, it is almost inevitable that the ER should be responsive to these pathways. Indeed, the need to adapt ER function to metabolic fluxes might have been the driving force for evolution of the UPR to begin with; the selective pressure was assuredly not a need to respond to agents like tunicamycin. With new methodologies for tracking metabolic fluxes and localization of redox-active metabolites, the linkages of these two processes can now be probed and unveiled.
